# Effects of pH on Olfactory Behaviours in Male Shore Crabs, *Carcinus maenas*

**DOI:** 10.3390/ani14060948

**Published:** 2024-03-19

**Authors:** Hannah Ohnstad, Amber Marie Jones, Bethany Howard, Paula Schirrmacher, Helga D. Bartels-Hardege, Jörg Detlef Hardege

**Affiliations:** Biological Sciences, School of Natural Sciences, Hull University, Hull HU6 7RX, UK; h.ohnstad-2017@hull.ac.uk (H.O.); amjones081997@gmail.com (A.M.J.); h.hardege@hull.ac.uk (H.D.B.-H.)

**Keywords:** climate change, ocean acidification, olfaction, behaviour, pheromone, shore crabs

## Abstract

**Simple Summary:**

Climate change potentially threatens biodiversity, and in a changing environment, it is vitally important that we learn to understand how animals react to the predicted changes. In marine organisms, the sense of smell governs almost all essential behaviours animals exhibit, from finding food and detecting a predator to finding a mating partner. Interpreting animal behaviour when exposed to odour is a complex task, as many factors from seasonality to individuality, fitness, social status, and even weather and water chemistry influence an individual’s response. Here, we examine the impacts of reducing seawater pH levels predicted for the end of the century upon decision-making in male shore crabs when exposed to the female reproductive odour, the female sex pheromone. There is a significant alteration in the responsiveness of male crabs, with large, more sexually active males taking significantly less time to detect and react to females but then showing less sexual mating activity once reaching the female odour. This disruption of olfactory communication can potentially impact the mating and reproductive success of this globally distributed species, showing that even coastal crustaceans that are known to be hardy and able to survive substantial stressors are potentially at risk from altered seawater chemistry associated with climate change.

**Abstract:**

The effects of climate change are becoming more apparent, predominantly concerning the impacts of ocean acidification on calcifying species. Many marine organisms rely on chemical signals for processes such as foraging for food, predator avoidance, or locating mates. The process of how chemical cues in marine invertebrates function, and how this sensory mode is affected by pH levels, is less researched. We tested the impact of reduced pH (7.6), simulating end-of-the-century predicted average ocean pH, against current oceanic pH conditions (8.2), on the behavioural response of male shore crabs *Carcinus maenas* to the female sex pheromone bouquet consisting of Uridine–diphosphate (UDP) and Uridine–triphosphate (UTP). While in current pH conditions (8.2), there was a significant increase in sexual interactions in the presence of female pheromone, males showed reduced sexual behaviours at pH 7.6. The crab weight–pH relationship, in which larger individuals respond more intensely sexually in normal pH (8.2), is reversed for both the initial detection and time to locate the cue. These results indicate that lowered pH alters chemical signalling in *C. maenas* also outside the peak reproductive season, which may need to be taken into account when considering the future management of this globally invasive species.

## 1. Introduction

Chemical communication is known as the language of life within the complex aquatic environment. Due to reduced visibility in turbid waters and the structurally complex habitat making visual cues less effective, organisms have adapted to use a wide variety of chemical signals and cues to instigate primary behaviours key to their survival [1]. Crustaceans have been extensively researched in relation to feeding stimulants, with many using low molecular weight metabolites like amino acids that are released from injured animals [2,3]. This complex mechanistic pathway used for communication is under threat from anthropogenic influences, including heavy metal pollution and climate change.

Over the last few centuries, the human population has rapidly expanded, causing many environmental issues, including a huge loss in biodiversity [4] and increased demand for dwindling marine resources, thus threatening food security [5,6]. This exponential growth has led to ecosystems changing locally and globally. The marine environment has been over-exploited [7], and the demand for fossil fuels has continued to grow, now exceeding 410 ppm [8]. Ocean acidification, caused by the increasing amount of CO_2_ within the atmosphere being absorbed by the oceans, could substantially alter marine ecosystems and have devastating impacts on the species within them [9].

The change in oceanic chemistry and temperature is proving to have a significant effect on our marine organisms, reducing their success in key behaviours such as feeding [10], predator/prey interactions, homing [11], and reproduction. The recent IPCC report 2022 [8] has shown that unprecedented and irreversible change, over hundreds and maybe thousands of years, has occurred in our climate, and without drastic action, the impacts of global warming will have long-reaching and severe impacts on the state of our oceanic environments. Water surface temperatures, by 2100, are predicted to increase 5–7 times more than compared to increases seen in the previous 50 years, with pH levels declining to 7.6 pH units by 2081–2100. The impacts of Ocean Acidification, caused by the decrease in pH levels, alter the chemical structure and function of molecules, impacting communication success [12].

The shore crab *Carcinus maenas* is a common inhabitant of various coastal habitats throughout Europe [13] and is used widely as a test organism in ecotoxicology [14,15]. Although *C. maenas* is native to areas around Europe and North Africa, in recent decades, it has invaded North America, Australia, parts of South America, and South Africa [16]. This crustacean is a successful invader due to its high tolerance to environmental perturbation [17] and is accustomed to environmental abiotic fluctuations due to its natural habitat, and many have made adaptations to counteract these stressors [18]. Therefore, shore crabs are widely used to study the impacts of environmental stressors on an organism’s physiology and the subsequent adaptations made to survive these extremes [16]. Shore crabs, like most marine invertebrates, rely on chemicals to communicate and evaluate their environment as well as coordinate key behaviours like feeding, reproduction, and predator detection [19].

Behavioural studies on sensory systems generally use one cue in isolation in clean, static tanks. However, in the environment, animals are exposed simultaneously to a variety of signals, such as the presence of prey/food, potential predators, and competitors, as well as mating partners. The best-studied potentially conflicting signalling systems are predator–prey interactions, where a range of impacts on olfactory cue-driven behaviours have been detected [20]. Since the chemical nature of many marine signals/cues is not elucidated yet, most studies on olfactory disruption also rely on unknown chemical cues from conditioned water or macerated food that cannot be quantified, making the interpretation of animal responses challenging. Interactions of competing signals, such as foraging for food vs. attraction to mating partners, also depend on the physiological state of the animals tested. In *Carcinus maenas*, this depends on the seasonality of the reproductive phase as well as social interactions [20]. Evaluating the readiness of individual animals to respond to an olfactory cue is essential to fully understand the impacts of stressors upon animal behaviour, but few studies address such complexities in their methods or data interpretation. This complexity in the interpretation of animal behaviour and the lack of quantifiable cues have been major contributors to the significant repeatability problem of previous studies on fish olfaction and ocean acidification, leading to controversial discussions about the impacts of OA on animal behaviour [21,22,23].

In this study, we examined how lower pH may affect a shore crab’s reaction to both natural and synthetic versions of the known female sex pheromone and food cues. We utilised flow-through Y-shape olfactometers with simulated additions at current pH (8.15) and predicted future (7.6) pH values. Examining multiple sensory signals simultaneously enables comparison of responses to competing signals at controlled concentrations of known, quantifiable chemical cues. Using marked individuals allows the inclusion of animal characteristics such as sex, size, and weight as a confounding variables to explain consequences to population dynamics resulting from pH change-driven shifts in behaviour. As such, we address here a gap in our knowledge of recent olfactory disruption literature [12,24].

## 2. Materials and Methods

Over 300 *Carcinus maenas* were collected by hand from natural seawater ponds surrounding the University of Algarve Marine Station (Ramalhete, grid coordinates: 37.006767945043585Ch-7.96741479703442), so transport time was minimal, and no casualties occurred. Male crabs were transferred into a large flow-through tank filled with water of pH 8.1, where they were kept under pH-controlled conditions for one week prior to experiments in 1.5 m × 1.5 m by 0.8 m height tanks, which held 1500 L, and they were fed with defrosted mussels (*Mytilus edulis*). The culture conditions were selected to mimic those in the estuary at the field station. The Ramalhete Marine Station (CCMAR, Faro, Portugal) was equipped with a constantly measuring direct CO_2_-control system adjusting pCO_2_. Two independent systems were used to control pH of 8.1 ± 0.015 and pH of 7.6 ± 0.008. For a measured total alkalinity of 2500 mmol/kg SW, the pCO_2_ was calculated as 537 and 1922 matm in the 8.1 and 7.6 pH water (CO_2_calc software v1.2.0). Seawater parameters were measured daily at 2.30 p.m. for temperature: 20.16 °C ± 1.05 °C, salinity: 35.67 PSU ± 0.26 PSU, and dissolved oxygen: 7.55 mg/L ± 0.16 mg/L (for details of methods, see [25,26]). These tanks contained tubes for the crabs to shelter in. The control tank pH was kept at pH 8.1 ± 0.015, and the reduced pH tank was kept at 7.6 ± 0.008. The total alkalinity of 2500 μmol/kg SW was measured, and the pCO_2_ was calculated as 537 and 1922 μatm for the control and reduced pH tank water, respectively (CO2calc software v1.2.0). Salinity, temperature, and dissolved oxygen were recorded as seawater parameters daily at 2.30 p.m. (mean temperature: 20.16 °C ± 1.05 °C, mean salinity: 35.67 PSU ± 0.26 PSU, mean dissolved oxygen: 7.55 mg/L ± 0.16 mg/L) [26]). The seawater was natural seawater pumped directly from the estuary at Ramalhete and cleaned via fluidised sand filters.

Females were transferred into a smaller (500 L) flow-through tank with pH 8.1 water and were kept under control conditions. Males and females were tested towards the end of the *Carcinus* reproductive season (late October) when most, but not all males should still be sexually active, and both sexes may also respond to feeding cues [2]. The carbonate chemistry of all water samples was determined from pH (measured with an Orion 8103SC pH electrode calibrated on the National Bureau of Standards (NBS) scale). The crabs were not fed throughout this experiment.

Sodium carboxymethylcellulose (medium viscosity, Sigma-Aldrich, Gillingham, U.K. C4888) was used to make gel with added mussel juice and/or synthetic pheromone/feeding cue compounds to achieve the test concentrations, as outlined below. Negative control gels were made with natural seawater, positive controls used crushed and 0.2 μm-filtered mussel juice, and two test gels used the synthetic feeding stimulant Glutathione 10^−4^ M [27] and the sex pheromone UDP/UTP 10^−4^ M at a ratio of 4:1 [28]. All chemicals were obtained from Sigma–Aldrich. These gels were freeze-dried and stored in a freezer (−20 °C) until required for testing. The cue diffusion rate was calculated prior to experimentation to ensure equal distribution of stimulants (see Supplementary Materials). This testing was carried out in October 2019.

### Experimental Design

**Figure 1:**
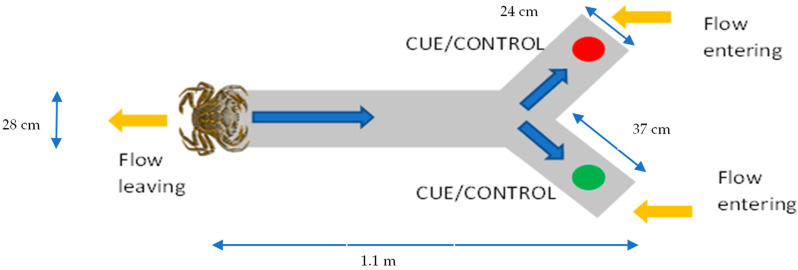
This graphic shows the setup of the Y-shaped olfactometer (length 1.1 m, base width 28 cm, arms 37 cm × 24 cm), with flow entering at the tips of the Y (yellow arrows) and flow leaving at the base of the olfactometer. Cue locations are indicated by green/red dots, and the crab movement options are indicated by the blue arrows. The crab is placed at the base before it is released. The crab in the figure is not to scale.

Two identical Y-shaped flow-through olfactometer tanks were set up with running natural seawater entering each branch of the tank with split flow tubes at a flow rate of 1 L per minute (Figure 1). Each tank was filled with seawater (20.16 °C ± 1.05 °C) to a depth of 12 cm; one tank was filled with water measured at pH 8.1 and the other at pH 7.6, modelling current and predicted ocean pH conditions for the year 2100. The pH was measured continuously throughout the study using an Orion 8103SC pH metre calibrated using the National Bureau of Standards (NBS) scale. Additionally, CO_2_ in the header tanks was continuously measured using an IRGA analyser (WMA-4; PP Systems, Amesbury, MA, USA) with data downloaded every 15 days. Salinity (CO310 conductivity metre; VWR, Radnor, PA, USA), pH (Orion 8103SC pH metre; Thermo Scientific, Waltham, MA, USA), temperature (Roth digital thermometer; Hanna Instruments, Woonsocket, RI, USA), and dissolved oxygen (Symphony SB90M5, VWR, Lutterworth, UK, accuracy ±0.2 mg/L; ±2%) were regularly monitored in the experimental aquaria. The tanks were positioned close together, so temperature and light intensity remained the same for both conditions. The tanks were lined with black liners to minimise external visual disturbances, such as shadows, that may distress the crabs or affect results [29,30]. The tanks were filled with 2.5 cm of sediment taken directly from the banks of the estuary and thoroughly rinsed with seawater to mimic natural environmental conditions. Silicone tea strainers were used to hold the cellulose gels in place at one end of the tank, enabling constant flow over the cues for controlled distribution (see Figure 1). The tanks were positioned outside and covered from above with mesh to create shade. Both sides of the tank were shaded evenly so no direct sunlight would enter past the mesh.

A preliminary test was carried out to assess the cue diffusion rate and how long the odour lasted until it was entirely diffused and could no longer be detected. The results of this showed that odours took approximately 5 min to diffuse to the other end of the Y-shaped tank at the chosen flow rate and lasted for a duration of approximately 2 h before needing to be replaced (Table S1). In the main study, crabs (n = 40 per condition, both sexes) were randomly selected from a large storage tank (pH 8.1). Control bioassays used a synthetic cue (Reduced Glutathione = GSH, or female sex pheromone = UDP/UTP) gel in one arm and a negative (seawater control) or a positive (mussel juice) gel in the other. This bioassay procedure was repeated for each experimental condition: GSH vs. seawater control, pheromone vs. seawater control, GSH vs. mussel juice, pheromone vs. mussel juice, and pheromone vs. GSH. Measurements of the crabs’ carapace width were taken using callipers (in cm) and recorded before they were placed into the tank. The size (CW–carapace width) of the *Carcinus* ranged from 1.5 to 8 cm, with crabs over 2 cm CW described as sexually mature in the Ria Formosa estuary [31]. The crabs were placed into a plastic tube in the tank and left for 2 min to allow them to acclimatise in the wake of the odours released from the gels. The tube was removed, allowing the crabs free movement. The time taken in seconds from the initial reaction of the crabs (time to initiate rapid antennule flicking) to them reaching the gel was recorded, and cue choice and behavioural observations (Table 1) in response to the cue were also recorded. The crabs were monitored for five minutes, if no movement was observed, then the crabs were removed and recorded as having no visible reaction. Antennule flicking was used as a behaviour indicating the detection of a feeding stimulant, as this has been commonly observed and reported in multiple decapod crustaceans [29]. This method was carried out by two observers in parallel, with one pH per tank ensuring all other environmental conditions were identical for each bioassay (such as time of day, light, weather). Data were recorded visually for each bioassay and put into Excel, where they were analysed using a combination of Excel and the statistical software RStudio 4.0.2. The crabs were released after testing as only natural odours had been used.

To analyse the differences between the time variables in each of the pH test conditions, we used the statistical software RStudio. The distribution of the data was checked via the Shapiro–Wilk test and inspected using histograms. As data were not normally distributed in some groups, non-parametric equivalents were used. The data for location and detection times were analysed using the unpaired two-samples Wilcoxon test (Wilcoxon rank-sum test). For the comparison of different behaviours in the two pH conditions, a generalised linear model (GLM) with a Poisson distribution was run, as the values being low in some areas meant that statistics, such as chi-squared, were unsuitable. Also, the data are dependent on each other as they were run in tandem to not add censorship to data that were compared together.

## 3. Results

### 3.1. Cue Location Time

**Figure 2:**
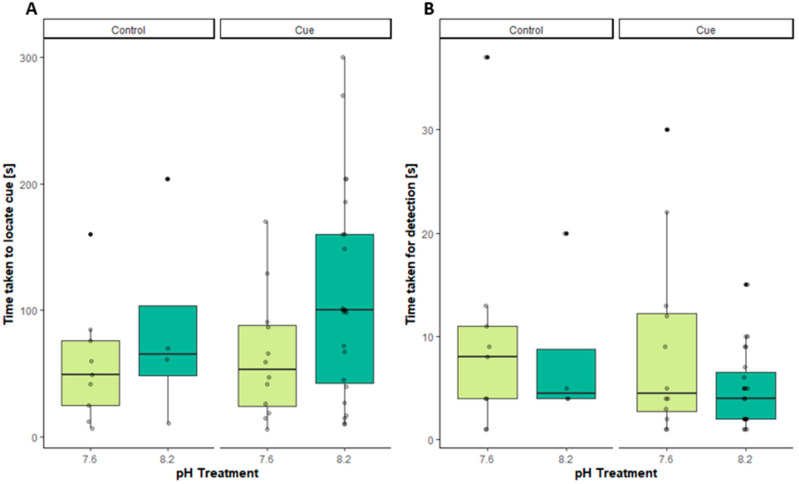
(**A**) Decision-making vs. responding at altered pH levels: time taken to locate cue choice by male *Carcinus maenas* reaching the end of the Y-shaped olfactometer arm in the choice of odour, either control or pheromone. Box plots represent the interquartile range. The boxplots depict the median with the first and third quartiles of the distribution. Whiskers extend to 1.5 times the interquartile range; data extending beyond this range are defined as outliers and plotted individually. (**B**) Time taken to initiate rapid antennae flicking by male *C. maenas* in response to chemical exposure (control and pheromone).

There was no significant effect of seawater pH (Wilcoxon rank-sum test with continuity correction, W = 88.5, *p*-value = 0.08844) (Figure 2A) on the time taken to locate the pheromone gel by the *Carcinus maenas*, as measured by the time taken to reach the end of the olfactometer arm after the crabs made a choice of odour (seawater or pheromone). There was no significant effect of seawater pH (Wilcoxon rank-sum test with continuity correction, W = 14, *p*-value = 0.5892) on the time taken to locate the control by *C. maenas*, as measured by the time taken to reach the end of the olfactometer arm after the choice of odour (seawater or pheromone), showing that pH does not influence locomotion ability.

### 3.2. Detection Time

There was no significant effect of seawater pH (Wilcoxon rank-sum test with continuity correction, W = 164.5, *p*-value = 0.3615) (Figure 2B) on the time taken to initiate rapid antennule flicking by *Carcinus maenas* that selected pheromone for the olfactometer choice (seawater or pheromone). There was no significant effect of seawater pH (Wilcoxon rank-sum test with continuity correction, W = 18, *p*-value = 1) (Figure 2B) on the time taken to initiate rapid antennae flicking by *C. maenas* that selected control for the olfactometer choice (seawater or pheromone), showing that pH has no impact upon the detection of the cues. The overall impact of pH on the induction of rapid flicking independent of the cue used follows the same pattern and is presented in the Supplementary Materials (Figure S1).

**Figure 3:**
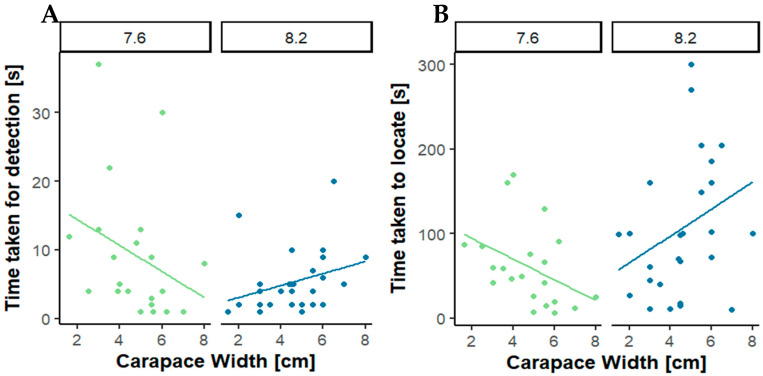
(**A**) Correlation between the time male crabs took to initiate antennal flicking as a measure of detection of a chemosensory cue, the female sex pheromone (UDP: UTP 4:1); (**B**). correlation between the time male crabs took to locate the cue, the female sex pheromone (UDP: UTP 4:1).

The responses of male crabs towards the pheromone are longer in normal pH and close to being significant (*p*-value = 0.08844, Figure 2A), and the variance in Figure 2A is greater at pH 8.2 than in reduced pH conditions. As experiments were undertaken towards the end of the reproductive season when not all male crabs respond to sex pheromones and larger, dominant males are more likely to respond to the female sex pheromone. We also analysed the data for a correlation of male size and the impacts of pH levels. Figure 3A,B shows marked size-dependent trends, both in the time taken to initiate antennal flicking and the time to locate a cue. While larger individuals under normal pH conditions take longer to detect and to locate the cue, this trend is reversed under low pH conditions.

**Figure 4:**
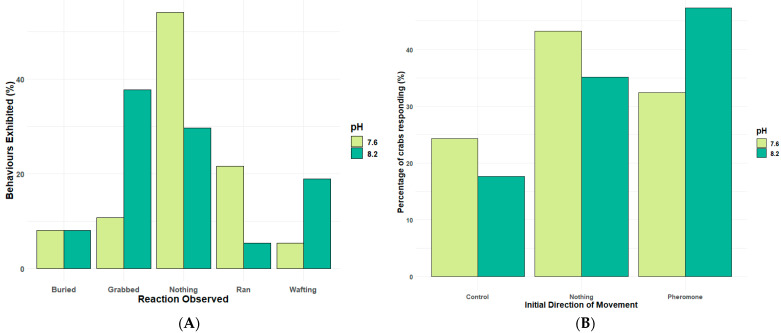
(**A**): Percentages of visual responses of different behaviours exhibited by male crabs exposed to female sex pheromone in a Y-shaped olfactometer. (**B**). The percentage of male crabs’ initial response in the direction selecting either not responding by doing nothing, selecting the pheromone, or the control baited arm of the Y-shaped olfactometer at current and future ocean pH levels. N = 74.

The mating behaviour of shore crabs involves a cascade of individual behaviour elements, from detecting a cue (antennal flicking), locomotion towards a cue, to grabbing and attempted mating or guarding. For definitions used here, see Table 1. A Poisson GLM was performed to compare the behaviours within the behavioural assays (Supplementary Materials, Figure S2). There was a significant effect of seawater pH (Estimate: 1.610 × 10^−15^, Std. Error: 0.388, z-value: 0.000, Pr (*p*-value): 1.000) (Supplementary Materials, Figure S2, Figure 4A) on these individual components of sexual mating behaviour exhibited by the *Carcinus maenas* in response to pheromone exposure.

There was a significant effect of seawater pH (Estimate: 1.099, Std. Error: 0.471, z-value: 2.331, Pr (*p*-value) 0.020 *) on the number of crabs exhibiting the full sexual mating behaviour, specifically the grabbing response, which is a key part of the mating process (Supplementary Materials, Figure S2). There was also a significant increase in the number of crabs exhibiting no visible behaviours at all in lower pH conditions (Estimate: 1.642, Std. Error: 0.446, z-value: 3.682, Pr (*p*-value): 0.000 **) (Supplementary Materials, Figure S2). There was no significant effect of seawater pH (Estimate: 0.511, Std. Error: 0.5164, z-value: 0.989, Pr (*p*-value): 0.323) on the number of crabs exhibiting the run behaviour, so crabs were still exploring the Y-shaped olfactometer (Supplementary Materials, Figure S2). There was no significant effect of seawater pH (Estimate: 0.406, Std. Error: 0.527, z-value: 0.769, Pr (*p*-value): 0.442) on the number of crabs exhibiting the wafting behaviour, which is associated with detecting a sexual cue by creating currents fanning a response signal towards a sender (Table 1; Supplementary Materials, Figure S2). Figure 4B shows that when looking at the decision-making of the male crabs to either stay at the starting area, run to the pheromone arm, or select the control arm of the olfactometer, there is a clear preference for the pheromone over the control at both pH levels; albeit, this is reduced in pH 7.6. At the same time, there is an increase in the number of males making no decision to run towards either chemosensory cue.

**Figure 5:**
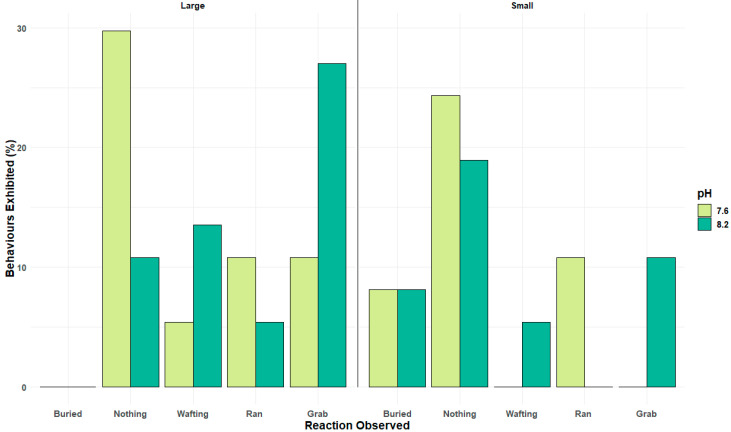
Percentages of visual responses of different behaviours exhibited by large and small males. Crabs exposed to female sex pheromone in a Y-shaped olfactometer at current and future ocean pH levels. N = 74.

When analysing the behavioural data shown in Figure 4A in relation to the size of the males, the effects of lowered pH become more pronounced (Figure 5) in the group of males that fall into a size class that has been described as sexually active.

## 4. Discussion

This study demonstrates that reduced ocean pH alters the chemosensory behaviour of the shore crab *C. maenas* [32]. Altering the detection and response to a chemosensory signal could have a multitude of potential reasons. These include the inability to detect the odour through receptor–ligand interaction disruption, as described for peptide cues [12], changes to the conformation of chemoreceptors [33], or alterations to signal transduction pathways such as GABA_A_ receptor alteration shown in a variety of fishes [22].

The hypothesis that the chemoreceptors for signalling compounds are affected by the reduced pH was proposed by [3]. They suggested that the increased hydrogen ions (H^+^) might alter the charge distribution on the odour receptor’s docking site of an animal’s sensory organs. Though this is difficult to test directly, it would reduce the ligand–receptor interactions, which affect signal detection on the same scale as changes to the signal molecule would [12,28]. Altered receptor signal interactions through both structural and charge distribution changes of the cue and the receptor were hypothesised as being responsible for altered signal detection [13]. Modelling of binding energies utilising known chemoreceptors enabled Schirrmacher et al. [33] to demonstrate the changes to the conformation of chemoreceptors for a predator cue in hermit crabs.

For the detection of a chemical signal, the cue must be available in a bioactive form above a detection threshold level, which is known to be impacted by the protonation status of the cues [13]. Lowered pH leads to higher abundance of the protonated form of a signalling molecule, here UDP/UTP, that then potentially impacts receptor–ligand interactions depending on the pKa of the compound. There was no significant difference between pH treatments for the control and the sex pheromone, suggesting that males can still detect pheromones in low pH at the same speed if they are delivered at a concentration of bioactive molecules above the detection threshold (Figure 2A). In fact, male crabs took slightly longer to reach the cue in the olfactometer at pH 8.2, showing that the physical ability to run towards a cue is not decreased in lowered pH conditions. The fact that pheromone-stimulated male crabs were able to reach the end of the arm slightly faster in low pH confirms (Figure 2A) shows that if the concentration of pheromone is high enough to be above the reaction threshold, a response is initiated regardless of pH. The data also suggest that there are no signs of either of a lack of neural stimulation, as described for fish and modification of GABA_A_ channels [22], or of metabolic depression caused by short-term low pH exposure. Equally, there was no significant increase in the time it took to exhibit antennule flicking from crabs tested in reduced pH compared to normal pH conditions, suggesting that low pH did not impair the crab’s ability to detect the odour (Figure 2B). At a very low pH of 6.6 [34], hermit crabs found impairments to olfactory behaviour, including antennular flicking and prey detection. However, these results are different to a study conducted on the freshwater crayfish, *Cambarus bartoni*, in which individuals showed a reduced rate of antennule flicking and failed to locate a food odour under low pH conditions [35].

Results from behavioural assays upon pheromone exposure depend on a diverse list of environmental, physiological, and social factors, making it difficult to quantify and compare studies [36]. This also includes the narrow window of cue concentration over which a response may shift from initiating a response to falling below the detection threshold. Even when the bioavailability of a cue is reduced by only a small percentage, dramatic effects can be recorded if the concentration of a bioactive cue falls below the detection threshold [36]. However, large changes in the bioavailability of the bioactive form of a cue may not result in any change in behavioural responses when they occur above the response threshold.

The detection of pheromones at lowered pH in *Carcinus maenas* is similar to what has been found for predator detection [20], which is independent of seawater pH. With shore crabs inhabiting coastal, often estuarine areas where pH conditions fluctuate significantly on a daily and seasonal level, the stability of key behaviours, especially towards potential lethal threats, is beneficial [21]. Nevertheless, the interpretation of male attraction in olfactometers is not always simplistic, as the reasons why an animal runs towards a cue can vary. When exposed to sexual stimuli, the observer usually assumes that the attraction is based upon the sexual cue. The attraction could potentially be based upon social interactions, gregariousness, or even cannibalism. Running fast may also not indicate attraction but could be escape behaviour, and altered pH along with altered odour cues could initiate a reaction of confusion, leading to crabs running away or hiding [32].

Interestingly, Figure 3 shows that the time to respond to a cue via increased antennal flicking and to locate are size-specific, with larger typically more sexually active males [32] taking longer to run towards the female cue than small males. Size, social hierarchies, and sexual maturity are also factors that could influence the results [36]. Perceived risk is also going to be different between larger and smaller crabs in a novel situation such as the bioassays. Smaller crabs, although having reached sexual maturity [31], do not respond as fast as larger, dominant male crabs that are known to win fights and respond quicker to sex pheromones [36]. Figure 3 also implies that large males select the olfactometer arm with the female pheromone. This relationship is completely reversed at decreased pH, supporting the hypothesis that decreased pH alters male mating attraction. This supports the interpretation that in sexually active males, reduced detection of female pheromones at reduced pH (see Figures S1 and S2 for responses of those males that selected the pheromone-baited arm of the Y-shaped olfactometer) could initiate a stress-type hypersensitive response when the sensory system is impaired [32]. Ovelheiro et al. [31] showed that the size at which male crabs reach sexual maturity differs between populations in Portugal and even more so on a wider geographical scale. Defining a size at which shore crabs are more sensitive to seawater pH levels is therefore too speculative and will require repeats at each population studied. Environmental adaptations like this are a major limiting factor in ecological research, highlighted recently to also impact the interpretation of behavioural assays [36].

Altered behavioural responses to cues could also show potential physical damage to chemoreceptors and sensory organs, as it has been shown that calcified animals may experience dissolution of their exoskeletons under such conditions [37,38,39] and may experience physiological stress, even though some responses may improve under reduced pH conditions [23,40,41]. Further testing found that the antennules of hermit crabs, after a five-day exposure to reduced pH seawater, did not reveal any visible damage when viewed under an electron microscope [42]. Similarly, Munday et al. [43] found no evidence of visible damage to the olfactory organs of their fish larvae using electron microscopy. However, Velez et al. [44] observed organisms developing mucus on the epithelia after exposure to low pH, presumably as a mechanism to protect themselves from the effects of low pH.

Whilst attraction towards an odour cue, such as the female sex pheromone, is a major element of the reproductive behaviour of many invertebrates and can be controlled by odour trails, Figure 4 highlights that the key step of forming a mating pair by grabbing the female or, in this case, the pheromone source, and attempting mating stance is impaired significantly by reduced pH, especially in those large sexually active *Carciuns* males (Figure 5). Pair formation, the mating stance, and post-mating guarding behaviour are critical to ensure successful reproduction in many brachyuran crustaceans. Altered or reduced success in low pH conditions is therefore a potentially significant impact of climate change-associated pH drop. In species that live in fluctuating pH conditions near the shore, such as estuarine environments where pH decreases significantly during the night and seasonally, the pH is lower in autumn/winter; such pH effects will be more pronounced in future oceans [45]. Our data could also be part of the reasons why some intertidal species, such as *C. maenas*, reproduce during the day in summer conditions when pH conditions are at their highest levels.

The fact that different elements of the complex reproductive behaviour are differently impacted by pH conditions in shore crabs also fits the reports by Richardson et al. [20] that predator detection is not altered by pH, while detection and response to food odours change in *Carcinus maenas* with experiments undertaken in the same location as the current study. Such complexity in the impacts of climate change is further increased when one considers the role of temperature and alkalinity in olfactory responses and their consequences for animal behaviour, as shown in a meta-analysis by Clements and Hunt [46]. Understanding olfactory disruption through climate change is therefore extremely difficult, as highlighted by the recent controversial discussion [22,23], and comparing studies on different species makes little sense ecologically [36]. To improve our understanding, there is a clear need to standardise our methods for this type of research to develop predictive models based upon the use of identified, quantifiable signalling compounds [36].

Overall, the percentile of males responding to the female sex pheromone was lower in this study than reported in previous studies [2,12]. This could be due to factors such as the pH, the stability of the cues, the smaller size of the males when used [32], as well as reproductive seasonality. Shore crabs’ mating season ranges between the months of April and October [32]. Male shore crabs in the UK show the highest responses to female sex pheromones in the month of July [12]. We conducted our research in October towards the end of the crabs’ reproductive season when the responsiveness to sex pheromones decreases significantly [26,27]. The cues were released from gels made from carboxycellulose that were frozen and then freeze-dried. The efficiency of pheromone cues in hermit crabs was not reduced due to freeze-drying [47,48].

Peptide signalling cues are susceptible to protonation in low pH conditions, altering the overall charge [12]. Peptide forms present today differ significantly from the protonated signalling peptides predicted in future oceans, including changes in molecular structure and electrostatic properties, which are crucial for receptor binding. Their study similarly used shore crabs, and results suggest an impaired functionality of the signalling peptides at low pH. The change of charge, structure, and consequently function of signalling molecules presents one possible mechanism to explain altered behaviour under future oceanic pH conditions [12]. The sex pheromone cue is a combination of two structurally similar, related nucleotides, UDP and UTP, and the structure of these two molecules changes shape and protonation under reduced pH albeit only by a small margin of less than 5% (Roggatz pers comm.). This is unlikely to render them significantly more difficult to detect by some of the shore crabs, but it reduces the bioavailability of pheromone in the correct, bioactive form slightly. Combined with the potential impacts of pH upon the structure and charge distribution at the chemoreceptor [28,33], this may leave some individual males impacted in their response to the pheromones.

For the duration of the study, male crabs were kept together in a large communal tank, meaning fights would occur. It is known that the shore crab changes its behaviour in relation to recent social interactions [23]. There was a large range in initial reaction times within the crabs, ranging from 0.01 s up to 60 s. These results could be in part explained by social status and hierarchy. Crabs of higher social status, typically larger crabs, win most fights and therefore will have a stronger response towards the female pheromone cue than smaller ‘submissive’ crabs with lower social status. Jiménez-Morales et al. [49] found that crabs remember their status after a fight, with the dominant and submissive male recognising their hierarchy status. Losers and winners will occur in all our tests; this randomisation allows the tests to be unbiased, and it may help explain the large variability in the data in terms of reaction time.

Kim et al. [34] found individual variation in the speed of antennule flicking and speed of prey detection, with crabs exposed to low pH treatments displaying higher individual variation than in the control pH treatment, suggesting that phenotypic diversity could promote adaptation to future ocean acidification. We similarly recorded a wider range of initial reaction times across the individuals tested in low pH treatments, ranging from 1– 70 s, supporting the suggestion that individual variation may relate to phenotypic diversity as a sign of adaptation to future ocean pH conditions. Individual variation in our study may have also been caused by additional factors such as social hierarchies (winner/loser effects, size), which can influence behavioural responses towards the cues [20,36].

Research related to cue alteration in the ocean, particularly animal responses to such changes, has shown that the cues themselves are directly influenced by pH [12,50]. Natural chemical cues are being modified by humans, and novel anthropogenic cues are being introduced into the ocean, both of which can directly and indirectly alter the persistence, composition, and transmission of natural cues. Natural cues can be either stable or unstable, whereas with synthetic cues, we can choose whether they are stable or not. When looking into synthetic cues and their uses in the future, it is important to test their effectiveness across varying levels of pH, including expected low pH levels. A reason for doing this is so that the cue will remain effective even amidst pH changes.

Limited research has been completed using gels infused with pheromone as a species-specific way to manage invasive species [51]. Although food cues may have a positive response on crabs, this odour would not be species specific to *C. maenas*, as many marine organisms are attracted to similar food odours, predominantly amino acids and peptides. *C. maenas* is one the most prolific marine invasive species, impacting commercial bivalve cultures as well as threatening the stability of ecosystems [52]. Our research provides insight into the complexity of utilising sex pheromones as a potential integrated pest management strategy in a changing world. It highlights the need for further research into the complex environmental and social behaviours of crustaceans that govern their interactions.

## 5. Conclusions

Low pH alters the responses to female sex pheromones in male shore crabs, with different elements of reproductive behaviour affected differently. The time to respond via antennular flicking and attraction to the source became more rapid in lowered pH, especially in large, sexually active males. This could be explained as hyperosmia due to olfactory stress in changed seawater chemistry, leading to heightened sensitivity to female odour. Males attracted to the source of the cue did show substantially decreased pheromone-induced mating activity, highlighting a potentially significant disruption of mating success.

The data show the complexity of how olfaction is impacted by pH conditions. This is especially important at the end of the spawning season when crabs’ responses to sexual cues are already reduced, as in our study. An individual’s size, physiology, moult stage, and potential social status impact their responses. This highlights that predicting the impacts of climate change-associated olfactory disruption accurately will require a much deeper understanding of such variables. There is a need to standardise our methods for this type of research to develop predictive models based on the use of identified, quantifiable signalling compounds.

## Figures and Tables

**Table 1:** This table describes the behaviours exhibited by *C. maenas* in the study.

Wafting	This behaviour can be defined by a rapid back-and-forth movement created by the *Carcinus maenas’* mouth pieces.
Grabbing	This behaviour was when the *Carcinus maenas* physically grabbed the tea strainer that the odour was inside of with either claw.
Buried	This behaviour was recorded if the *Carcinus maenas* buried into the sediment, either at the start of the experiment or near a cue.
Non-Visible	This behaviour was recorded if the *Carcinus maenas* didn’t show any visible behaviours.
Detection	This behaviour was defined by the onset of the *Carcinus maenas* rapidly flicking their antennules.
Locating	This behaviour was defined by whether the *Carcinus maenas* made a decision and reached the end of one of the Y-shaped olfactometer arms
Cradle cue	This behaviour was recorded when the *Carcinus maenas* showed cradling behaviour towards the cue.
Ran at cue	This behaviour was recorded if the *Carcinus maenas* reached the cue and then continued to run in a confused manner around it.

## Data Availability

Data are available upon request.

## Supplementary Materials

The following supporting information can be downloaded at: https://www.mdpi.com/article/10.3390/ani14060948/s1, Figure S1. Time taken to initiate rapid antennae flicking (±SE) by male *Carcinus* in response to chemical odours at reduced and normal pH levels. Control (blank control gel) and sex pheromone are grouped, as all animals were exposed to odours for detection rates to show the impacts of pH on animal responsiveness to odours. Figure S2. Time taken to initiate rapid antennae flicking (±SE) by male *Carcinus* in response to pheromone for those selected to go down the pheromone arm on the olfactometer at reduced and normal pH levels. Figure S3. Time taken to reach the end of an olfactometer arm by male *Carcinus* in response to pheromone for those selected to go down the pheromone arm on the olfactometer at reduced and normal pH levels. Table S1. Time for odour cues to reach the end of the Y-shaped olfactometer. Table S2. Poisson GLM output, N = 37.
