# Supplementary material for: Effects of pH on Olfactory Behaviours in Male Shore Crabs, Carcinus maenas
Source: Animals (Basel). 2024 Mar 19;14(6):948. doi: 10.3390/ani14060948 (PMC10967494; doi:10.3390/ani14060948)
Supplement: Supplementary file 1 [file animals-14-00948-s001.zip › animals-2824325-supplementary.pdf]

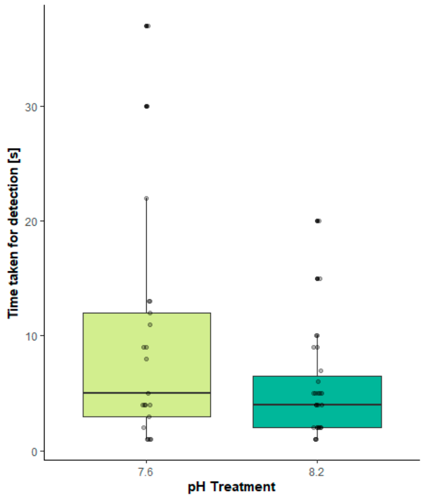

Figure S1. Time taken to initiate rapid antennae flicking (± SE) by male *Carcinus* in response to chemical odours at reduced and normal pH levels. Control (blank control gel) and sex pheromone are grouped as all animals were exposed to odours for detection rates to show impacts of pH on animal responsiveness to odours.

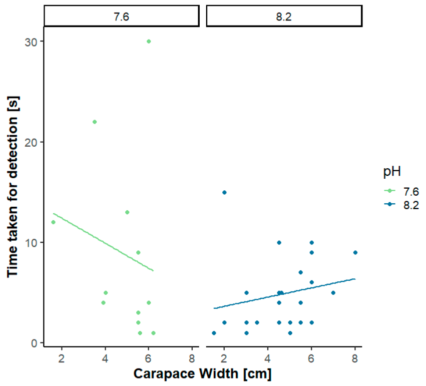

Figure S2. Time taken to initiate rapid antennae flicking (± SE) by male *Carcinus* in response to pheromone for those that selected to go down the pheromone arm on the olfactometer at reduced and normal pH levels.

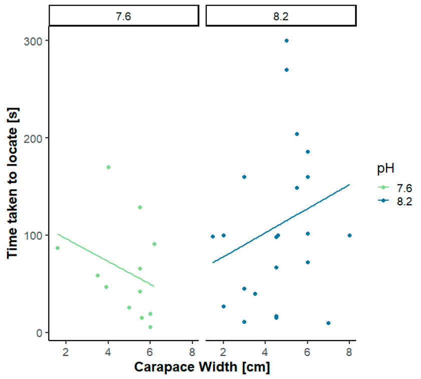

Figure S3. Time taken to reach the end of an olfactometer arm by male *Carcinus* in response to pheromone for those that selected to go down the pheromone arm on the olfactometer at reduced and normal pH levels.

Table S1. Time for odour cues to reach the end of the Y-shaped olfactometer.

**Flow Rate**	**Time to diffuse (Minutes)**
0.5 L/Min	9.14
1 L/Min	5.01
1.5 L/Min	3.45
2 L/Min	2.46
2.5 L/Min	2.01
3 L/Min	1.32
3.5 L/min	0.54
4 L/Min	0.36

Table S2. Poisson GLM output, N=37.

	Estimate	Std. Error	z value	Pr (p-value)
(Intercept)	1.0099	3.088	0.3566	0.722
pH	1.610 e-15	0.388	0.000	1.000
Grabbed	1.099	0.471	2.331	0.020 *
Nothing	1.642	0.446	3.682	0.000***
Ran	0.511	0.5164	0.989	0.323
Wafting	0.406	0.527	0.769	0.442
